# “Slow” Voltage-Dependent Inactivation of CaV2.2 Calcium Channels Is Modulated by the PKC Activator Phorbol 12-Myristate 13-Acetate (PMA)

**DOI:** 10.1371/journal.pone.0134117

**Published:** 2015-07-29

**Authors:** Lei Zhu, Sarah McDavid, Kevin P. M. Currie

**Affiliations:** 1 Department of Anesthesiology, Vanderbilt University, Nashville, Tennessee, United States of America; 2 Department of Pharmacology, Vanderbilt University, Nashville, Tennessee, United States of America; 3 Vanderbilt Brain Institute, Vanderbilt University, Nashville, Tennessee, United States of America; Dalhousie University, CANADA

## Abstract

Ca_V_2.2 (N-type) voltage-gated calcium channels (Ca^2+^ channels) play key roles in neurons and neuroendocrine cells including the control of cellular excitability, neurotransmitter / hormone secretion, and gene expression. Calcium entry is precisely controlled by channel gating properties including multiple forms of inactivation. “Fast” voltage-dependent inactivation is relatively well-characterized and occurs over the tens-to- hundreds of milliseconds timeframe. Superimposed on this is the molecularly distinct, but poorly understood process of “slow” voltage-dependent inactivation, which develops / recovers over seconds-to-minutes. Protein kinases can modulate “slow” inactivation of sodium channels, but little is known about if/how second messengers control “slow” inactivation of Ca^2+^ channels. We investigated this using recombinant Ca_V_2.2 channels expressed in HEK293 cells and native Ca_V_2 channels endogenously expressed in adrenal chromaffin cells. The PKC activator phorbol 12-myristate 13-acetate (PMA) dramatically prolonged recovery from “slow” inactivation, but an inactive control (4α-PMA) had no effect. This effect of PMA was prevented by calphostin C, which targets the C1-domain on PKC, but only partially reduced by inhibitors that target the catalytic domain of PKC. The subtype of the channel β-subunit altered the kinetics of inactivation but not the magnitude of slowing produced by PMA. Intracellular GDP-β-S reduced the effect of PMA suggesting a role for G proteins in modulating “slow” inactivation. We postulate that the kinetics of recovery from “slow” inactivation could provide a molecular memory of recent cellular activity and help control Ca_V_2 channel availability, electrical excitability, and neurotransmission in the seconds-to-minutes timeframe.

## Introduction

Ca_V_2.2 (N-type) voltage-gated calcium channels (Ca^2+^ channels) are widely expressed in neurons and neuroendocrine cells where they control neurotransmitter / hormone secretion, gene expression, activation of Ca^2+^-dependent enzymes / ion channels, and a variety of other cellular functions. Calcium entry is precisely regulated by second messengers including G proteins, kinases, and lipid signaling molecules that converge to fine tune Ca_V_2 function [1–8]. Ca^2+^channel inactivation also controls Ca^2+^ entry and thus cellular excitability and short term synaptic plasticity [9–12]. Ca_V_2 inactivation is mediated by distinct calcium or voltage-dependent mechanisms: calcium-dependent inactivation is triggered by “global” elevations of cytosolic [Ca^2+^] and transduced via calmodulin tethered to the C-terminal tail of the channel [13–17]. Voltage-dependent inactivation is complex, can occur from both the open and closed states of the channel, and exhibits multiple kinetic components in response to sustained or repetitive membrane depolarization. “Fast” inactivation (onset / recovery from tens—hundreds of milliseconds) is thought to involve a “hinged-lid” type pore occlusion by the cytoplasmic loop linking the first and second domains of the α1 subunit (the I-II linker) [11, 18, 19]. The auxiliary β subunit of the channel binds this I-II linker and modulates the kinetics of “fast” inactivation [20, 21], as do heterotrimeric G protein βγ subunits (Gβγ) [22]. An additional inactivated state, revealed by sustained membrane depolarization, displays much slower onset and recovery kinetics (seconds-to-minutes range) [23–25]. “Slow” inactivation is also found in potassium and sodium channels and might involve changes in the voltage-sensor domain and/or constriction of the channel pore [26–29].

Interestingly, protein kinases modulate “slow” inactivation of sodium channels and thereby control neuronal excitability [30, 31]. Much less is known about how “slow” inactivation of Ca_V_2 channels is regulated. The Ca_V_ β subunit might play a role as an indirect consequence of altered “fast” inactivation [24], and syntaxin has been reported to promote “slow” inactivation of Ca_V_2.2 [25, 32]. In this study we show for the first time that phorbol ester (PMA) dramatically prolongs recovery of Ca_V_2 channels from “slow” inactivation. We postulate this novel regulation could provide a basis for molecular memory of recent cellular activity and help control Ca^2+^channel availability, electrical excitability, and neurotransmission in the seconds-to-minutes timeframe.

## Materials and Methods

### Cell culture and transfection

Recombinant channels were recorded from transiently transfected HEK293 cells or from G1A1 cells (HEK293 cells stably expressing Ca_V_2.2, β_1b_, and α_2_d subunits) kindly provided by Dr. Heidi Hamm (Vanderbilt University) [22, 33, 34]. Transient transfection with Qiagen purified plasmids (Valencia, CA) was performed using lipofectamine 2000 (Invitrogen, Grand Is., NY) in 35mm tissue culture dishes as *per* manufacturer instructions. Cells were transfected with calcium channel subunits in a ratio of 1;1;1 (Ca_V_2.2, α_2_δ, and either β_1b_ or β_2a_). The β subunit plasmid also expressed EGFP downstream of an IRES sequence to enable visual identification of transfected cells. In some experiments cells were transfected with Ca_V_2.1, β_2a_ and α_2_δ. The specific contructs used were as follows: Ca_V_2.1, rat α_1A_ subunit (Genbank # M64373) and rat α_2_δ Genbank # M86621) both kindly provided by Dr Terry Snutch (University of British Columbia, Vancouver, Canada); Ca_V_2.2 –bovine α_1B_ (Genbank # NM174632) and bovine β_1b_ (Genbank # AF174415) both kindly provided by Dr Aaron Fox (University of Chicago, Chicago IL); rat brain β_2a_ (Genbank # M80545) kindly provided by Dr Roger Colbran (Vanderbilt University, Nashville TN). Patch-clamp recording was performed ~48–72 hours after transfection on cells that had been re-plated onto poly-lysine coated glass coverslips for ~12–24 hours. Transfected cells were visually identified using fluorescence of EGFP. The culture medium consisted of MEM supplemented with fetal bovine serum (10%), glutamine (2mM), penicillin/streptomycin (100 unit ml^-1^/100 μg ml^-1^), and for G1A1 cells the medium also included G418 (0.5 mg ml^-1^). Cells were maintained in an incubator (37 C in 95% air and 5% CO_2_ at ~90% humidity) and passaged every 3–5 days for up to ~25 passages.

Adrenal chromaffin cells: Male bovine adrenal glands were obtained from a local slaughterhouse (C & F Meat Co. Inc., College Grove, TN), and chromaffin cells prepared by digestion with collagenase followed by density gradient centrifugation as described previously [35]. The cells were plated onto collagen-coated coverslips at a density of ~0.2 x 10^6^ cell /mL. Fibroblasts and other proliferating cells were effectively suppressed with cytosine arabinoside (10 μM) (Sigma-Aldrich; St Louis MO), leaving relatively pure chromaffin cell cultures. The culture medium for chromaffin cells consisted of Dulbecco’s modified Eagle medium \ F12 (1:1) supplemented with fetal bovine serum (10%), glutamine (2 mM), penicillin/streptomycin (100 unit mL^-1^/100 μg mL^-1^), cytosine arabinoside (10 μM) and 5-fluorodeoxyuridine (10 μM). The culture medium was replaced the day after isolation and experiments were performed 2–5 days following cell isolation. All tissue culture reagents were from Life Technologies (Grand Island, NY).

### Patch-clamp electrophysiology

Cells were placed in a recording bath (volume ~300 μL) which was continually perfused with fresh solution at a flow rate of ~3–4 ml/min from gravity-fed reservoirs, and viewed using a Nikon TE2000 inverted microscope. Patch pipette electrodes were pulled from borosilicate glass capillary tubes (World Precision Instruments, Sarasota, FL) using a Sutter P-97 pipette puller (Sutter Instruments, Novato, CA), coated with dental wax (Electron Microscopy Sciences, Hatfield, PA) and fire-polished to a final resistance of ~2 MΩ when filled with a CsCl-based internal solution. Cells were voltage-clamped in the conventional whole-cell configuration using an Axopatch 200B amplifier, Digidata 1400A interface, and PClamp10 (Clampex) acquisition software (Molecular Devices, Sunnyvale, CA). Analog data were filtered at 2–3 kHz and digitized at 50 kHz. Series resistance was partially compensated using the Axopatch circuitry (~60–80%). Linear capacitance and leak subtraction (performed offline) used P/N protocols (P/-4 or P/-8) with the leak pulses applied following the test pulses. Some of the voltage-protocols involved very long stimulus steps or trains. For these experiments leak subtraction was not applied, in part because the protocols were designed to monitor recovery from inactivation which could be altered in a voltage-dependent manner. In these experiments only cells with high resistance seals (> 1GΩ) and low holding current (< 50 pA) that remained stable for the duration of the experiment were used. Raw data were analyzed using PClamp10 (Clampfit) and graphing / statistical analyses were performed using OriginPro software (Originlab Corporation, Northampton, MA) or Prism5 software (GraphPad Software Inc., La Jolla, CA). All experiments were performed at room temperature (~ 23–25°C).

### Solutions, drugs and reagents

The intracellular (patch pipette) solution consisted of (in mM): CsCl 110, MgCl_2_ 1, HEPES 20, BAPTA 10 (sodium salt), Na_2_GTP 0.35, adenosine triphosphate (MgATP) 4, creatine phosphate (sodium or tris salt) 14, pH 7.3, osmolarity ~310–315 mOsm. In some experiments GTP was omitted and 0.5 mM GDP-β-S (lithium salt) was included to test the potential involvement of G protein signaling. In other experiments a peptide inhibitor of PKC (2μM PKC(19–36)) or a peptide inhibitor of dynamin (50 μM dynamin inhibitory peptide) were added to the patch pipette solution on the day of use.

The extracellular NaCl-based solution used to bathe cells before and during seal formation consisted of (in mM): NaCl 145, KCl 2, MgCl_2_ 1, glucose 10, HEPES 10, CaCl_2_ 2, pH 7.3, osmolarity approx 315 mOsm. After entering the whole-cell recording configuration the bath solution was switched. For HEK293 and G1A1 cell recordings the extracellular solution contained (in mM): tetraethylammonium Cl 155, glucose 10, HEPES 10, BaCl_2_ 5, pH 7.3, 320-330mOsm. For chromaffin cell recording it contained (in mM): NaCl 150, KCl 2, MgCl_2_ 2, glucose 10, HEPES 10, CaCl_2_ 5, TTX 0.05–0.1, pH 7.3, osmolarity approximately 315 mOsm. Note barium and TEA were not used in chromaffin cells recordings because they both block potassium channels which results in depolarization of all the non-voltage-clamped cells in the recording chamber. These cells release a variety of neurotransmitters and hormones which can alter the cell being recorded from, for example via G protein coupled receptors [36].

Tetrodotoxin (TTX) (R&D systems, Minneapolis, MN) was prepared as a 1 mM aqueous stock and aliquots frozen until use (final concentration when diluted into extracellular solution was ~0.5 μM). PMA, 4α-PMA (Sigma-Aldrich, St Louis, MO) bisindolylmaleimide-1, Go6983 and calphostin C (R&D systems, Minneapolis, MN) were all prepared as stock solutions in DMSO (1-2mM) and aliquots diluted in extracellular solution on the day of use (final concentration of DMSO was 0.01–0.05%).

### Statistical analyses

Statistical analyses were performed using OriginPro software (Originlab Corporation, Northampton, MA) or Prism5 software (GraphPad Software Inc., La Jolla, CA). Recovery from inactivation was fit with a single or double exponential association function of the form: Y = Y_0_ + A(1-e^-X/t^) or Y = Y_0_ + A_1_(1-e^-X/t1^) + A_2_(1-e^-X/t2^) in which Y_0_ is the offset / initial amplitude at recovery time zero, A is the amplitude or span, X is the recovery time and t is the recovery time constant. For representation in figures, the mean recovery data were plotted and then fit with the above equations. The parameters from the fits to the mean data are reported in the figure legends. For statistical comparison of specific fit parameters (i.e. recovery time constants), each cell was individually fit with the exponential function to yield the relevant parameters and these were then pooled for statistical comparison. Statistical significance between two datasets was determined using paired or independent Student’s t-test, and ANOVA was used to compare multiple datasets.

## Results

### PMA selectively targets recovery from “slow inactivation”

**Fig 1:**
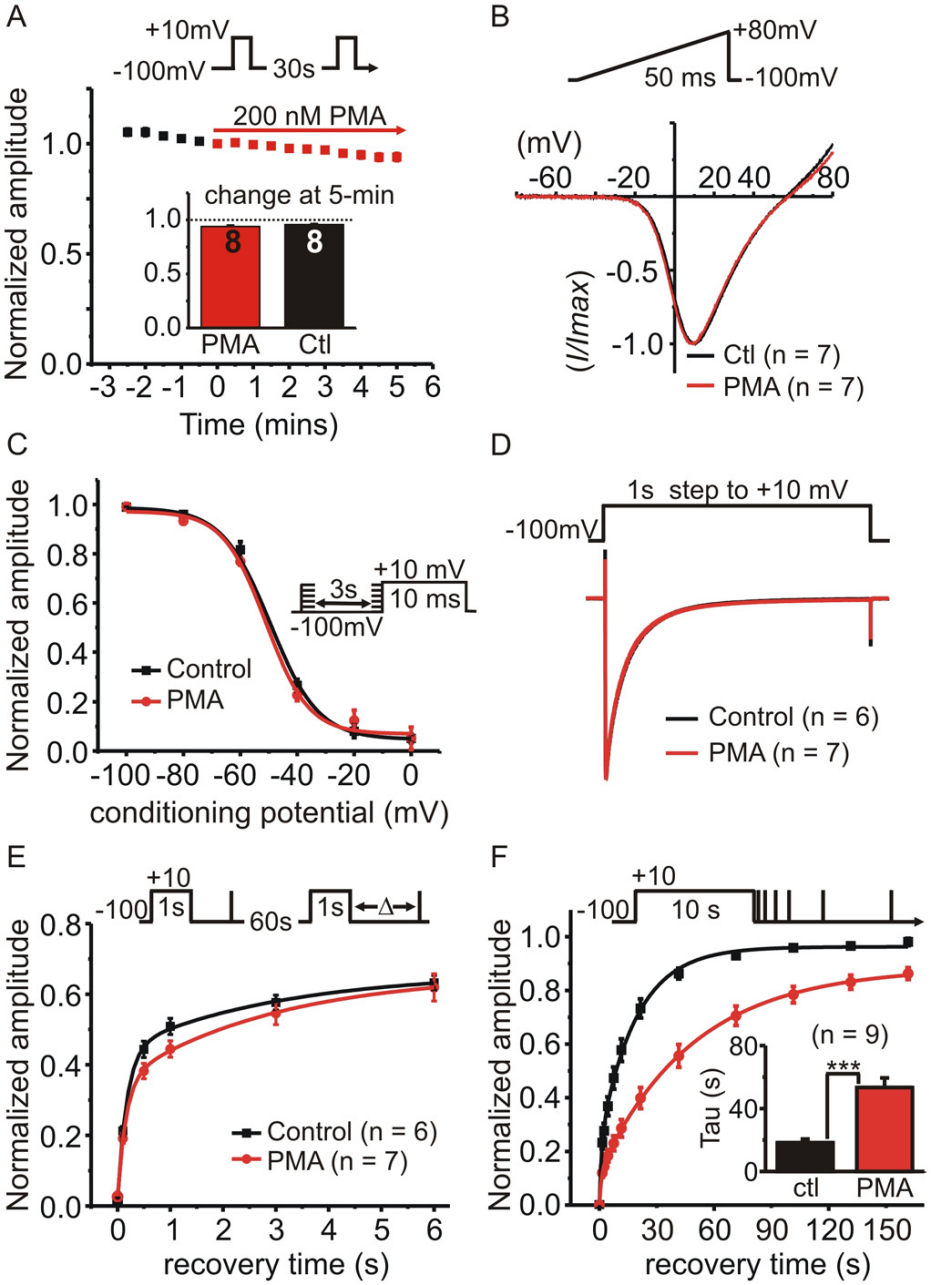
Phorbol ester (PMA) selectively targets recovery from “slow” inactivation. The effects of acute application of PMA on HEK293 cells expressing Ca_V_2.2, β_1b_ and α_2_δ were investigated. **(A)** Cells were stimulated with a 10ms step depolarization and peak amplitude of *I*_*Ba*_ plotted against time (normalized to the time point immediately before PMA application) (n = 8; error bars are plotted but in most cases fall within the symbol so are not visible). The inset bar graph shows the change in *I*_*Ba*_ amplitude after 5-minutes of PMA (200 nM) was not different from time-matched control cells. **(B)** Shows the normalized current-voltage relationship of *I*_*Ba*_ evoked by a ramp depolarization first in the absence (Ctl) then in the presence of PMA (200 nM for 5-min). Traces are the mean values (n = 7) and error bars are omitted for clarity. **(C)** The voltage-dependence of closed-state inactivation was determined before and during application of PMA (protocol shown in the inset). The mean normalized current amplitude was plotted and fit with a Boltzmann function of the form: I = I_2_ + (I_1_ —I_2_) / 1 + e((V—V_50_)/k)). The two curves (control and PMA) were not significantly different from one another (F = 0.97 p = 0.45); V_50_ = -49 mV in control and -51 mV in PMA, slope = -7.38 in control and -7.4 in PMA. **(D)** Inactivation of *I*_*Ba*_ during a 1s step depolarization was not altered by PMA. Control cells or cells treated with PMA for 5–10 minutes were stimulated with a 1s step to +10mV and the evoked currents normalized to the peak amplitude to enable better comparison of the inactivation time-course. Traces show the means but error bars are omitted for clarity. **(E)** Recovery from “fast” inactivation was not significantly different in control cells or PMA treated cells (200nM for 5–10 minutes). Inactivation of *I*_*Ba*_ was produced by 1s prepulse and recovery determined by a brief test pulse after the indicated interval. This was repeated once every 60s (see inset above graph for voltage-protocol). Current amplitude during the recovery test pulse was normalized to peak *I*_*Ba*_ evoked by the prepulse. The solid lines show double exponential fits to the data. **(F)** PMA prolonged recovery from “slow inactivation”. Inactivation of *I*_*Ba*_ was produced by 10s prepulse and recovery was tracked using a series of brief test pulses applied at the indicated time points following the 10s prepulse (see inset). This was repeated twice in the same cell, once before application of PMA ((Ctl) and once in the presence of PMA (after 5-min exposure). Solid lines show double exponential fits to the mean data (control A_1_ = 0.23, A_2_ = 0.73, t_1_ = 1.21 s, t_2_ = 18.96 s; PMA A_1_ = 0.12, A_2_ = 0.76, t_1_ = 1.06 s, t_2_ = 49.2 s, fit comparison F = 27.6 p < 0.0001). The inset bar graph shows the mean time-constant for the slow phase of recovery calculated from fits to the individual cells (*** p = 0.001, paired t-test).

We set out to investigate voltage-dependent inactivation of Ca_V_2.2 Ca^2+^ channels and how it might be regulated by the PKC-activator PMA. To isolate voltage-dependent inactivation, we used barium rather than calcium as the extracellular divalent cation and included BAPTA (10 mM) in the intracellular patch-pipette solution to prevent calcium-dependent inactivation. G1A1 cells stably express Ca_V_2.2, α_2_δ and β_1b_ calcium channel subunits. Under our recording conditions, acute application of PMA (200 nM for 5-minutes) had no effect on the peak barium current (*I*_*Ba*_) amplitude (Fig 1), and did not shift the current-voltage-relationship (Fig 1B), the voltage-dependence of inactivation (Fig 1C), or the rate/extent of inactivation during a 1s step depolarization (Fig 1D). To assess recovery of *I*_*Ba*_ from “fast” inactivation, we used a standard protocol in which a 1s prepulse (used to produce inactivation) was followed by a short test pulse to determine the extent of recovery (Fig 1E). This double pulse protocol was repeated every 60s and the recovery interval following the prepulse increased with each stimulus. Recovery following the 1s step was relatively fast (~ 50% recovery within 1s). Due to concerns with rundown and the possibility of a much slower component to the recovery, we limited this protocol to five repeats, covering the first 6-seconds of recovery. These data were fit well with a double exponential (Fig 1E) and neither of the fitted time constants were significantly changed in PMA treated cells (tau fast = 167 ± 12 ms in controls Vs. 161 ± 6 ms in PMA treated cells, p = 0.67 unpaired t-test: tau slow = 2.46 ± 0.49 s in controls Vs 3.1 ± 0.21 s in PMA treated cells, p = 0.23 unpaired t test).

To investigate recovery from “slow” inactivation we used a protocol consisting of a prepulse lasting 10 s that was followed by a series of 12 brief steps (8ms duration) applied at increasing intervals following the prepulse (Fig 1F). It was immediately apparent that recovery was slower following the 10s prepulse compared to that following the 1s prepulse. Recovery was best fit with a double exponential with a smaller fast and larger slow component (fast component = 20 ± 2% in control conditions and 13 ± 2% in PMA, n = 9, p = 0.02, paired t-test). The fast time constant was not significantly altered by PMA (890 ± 146 ms in control and 914 ± 108 ms in PMA, p = 0.84 paired t-test), but the slower time constant was dramatically prolonged from 18.3 ± 2.3 s in control conditions to 53.4 ± 6.1 s in the presence of PMA (n = 9, p = 0.0001, paired t-test). These data show that, under our recording conditions, PMA selectively targets recovery from “slow” inactivation with little or no effect on other parameters of *I*_*Ba*_.

### Recovery from “slow inactivation” is influenced by both the Ca_V_β subunit and by PMA

**Fig 2:**
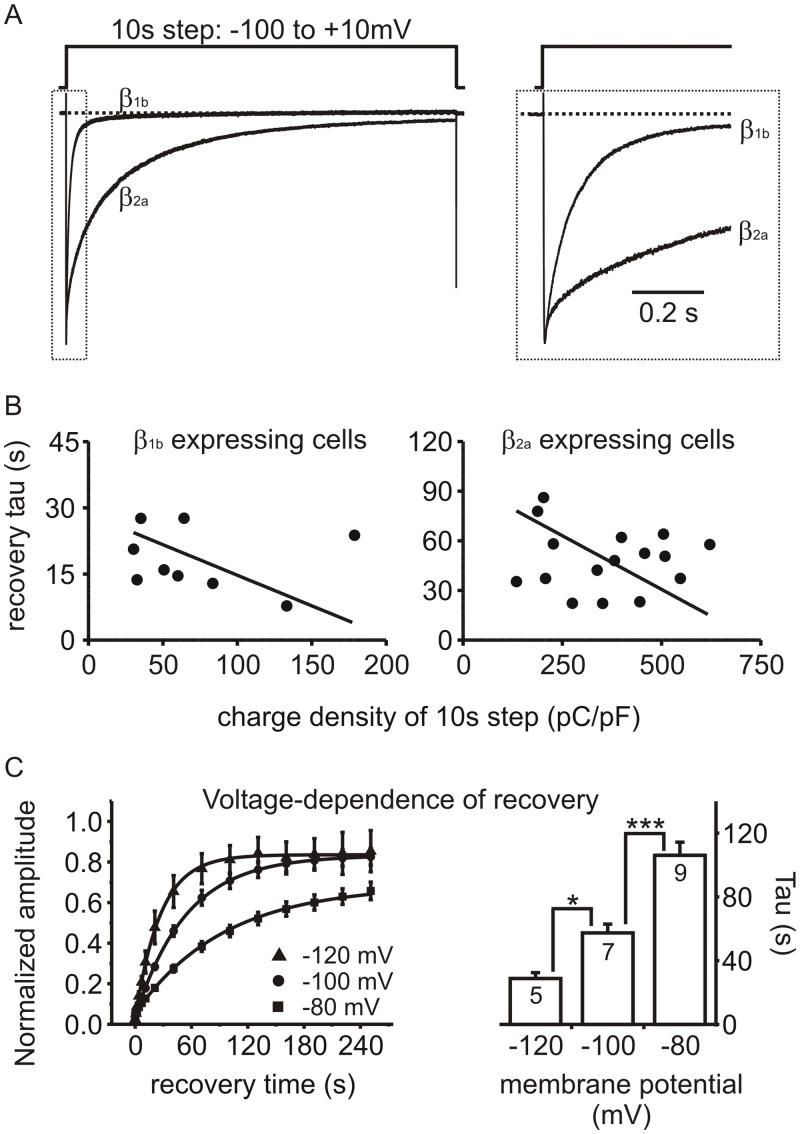
Recovery of Ca_V_2 channels from “slow” inactivation: dependence on voltage and Ca_V_ β subunit, but not charge density. **(A)** Representative *I*_*Ba*_ from HEK293 cells transfected with Ca_V_2.2, α_2_δ, and either β_1b_ or β_2a_ subunits. In both cases inactivation was almost complete by the end of the 10s step, but the β_2a_ subunit dramatically reduced “fast” inactivation. The right panel shows the initial ~600ms of the traces on an expanded time base. **(B)** Recovery rate was not correlated with barium entry. Inactivation was produced by a 10s prepulse and recovery tracked as in Fig 1F. The recovery time constant (tau) is plotted against charge density (i.e. the integral of *I*_*Ba*_ during the 10s prepulse normalized to cell capacitance. The solid line shows a linear fit using Deming regression. The slope of this line was not significantly different from zero (p = 0.75 for β_1b_ expressing cells and p = 0.76 for β_2a_ expressing cells). **(C)** Recovery from inactivation was voltage-dependent. Inactivation of β_2a_ containing channels was produced by a 10s prepulse and recovery tracked at different holding potentials (-80 mV, -100 mV, or -120 mV). Solid lines show exponential fits to the mean data (-120mV holding A = 0.79, t = 24.8 s; -100mV holding A = 0.8, t = 54.7 s; -80 mV holding A = 0.62, t = 103.2 s). The bar graph plots the mean time constant calculated from fits to the individual cells (* p < 0.05, *** p < 0.001 using ANOVA and Tukey’s post-test for pairwise comparisons).

Given that PMA appeared to target recovery from “slow” but not “fast” inactivation, we investigated the effects of PMA on channels containing the β_2a_ subunit which dramatically reduces fast inactivation [20, 21]. HEK293 cells were transiently transfected with Ca_V_2.2, α_2_δ, and either β_2a_ or β_1b_ subunits (with EGFP as a marker). As expected, inactivation of *I*_*Ba*_ during a step depolarization was much slower in β_2a_ than β_1b_ containing channels (Fig 2A). Recovery of β_2a_ channels was fit with a single exponential with a time constant (at a holding potential of -100 mV) of 48.6 ± 4.7 s (n = 16), which was significantly longer compared β_1b_ containing channels (19.5 ± 1.8 s; n = 4; p = 0.0007 unpaired t-test). As expected for *voltage-dependent* inactivation, the recovery kinetics of *I*_*Ba*_ showed no correlation with the amount of barium entry (Fig 2B). The overall amount of barium entry (charge density) was much greater in cells expressing the β_2a_ subunit than in G1A1 cells (β_1b_ expressing cells). However, when the recovery time constant was plotted against charge density, the slope of a linear fit using Deming regression was not significantly different from zero (0 = 0.75 for β_1b_ expressing cells and p = 0.76 for β_2a_ expressing cells). This was confirmed using Pearson’s correlation coefficient which again showed no statistically significant correlation between recovery rate and charge density: for β_1b_ cells r = -0.13, p = 0.74; for β_2a_ cells r = -0.08, p = 0.76. We also found that, as predicted for voltage-dependent inactivation, recovery was significantly accelerated at more hyperpolarized holding potentials (Fig 2C). Thus, recovery following the conditioning prepulse demonstrated the expected features for voltage-dependent inactivation, and the recovery kinetics were influenced by the subtype of the Ca_V_β subunit.

**Fig 3:**
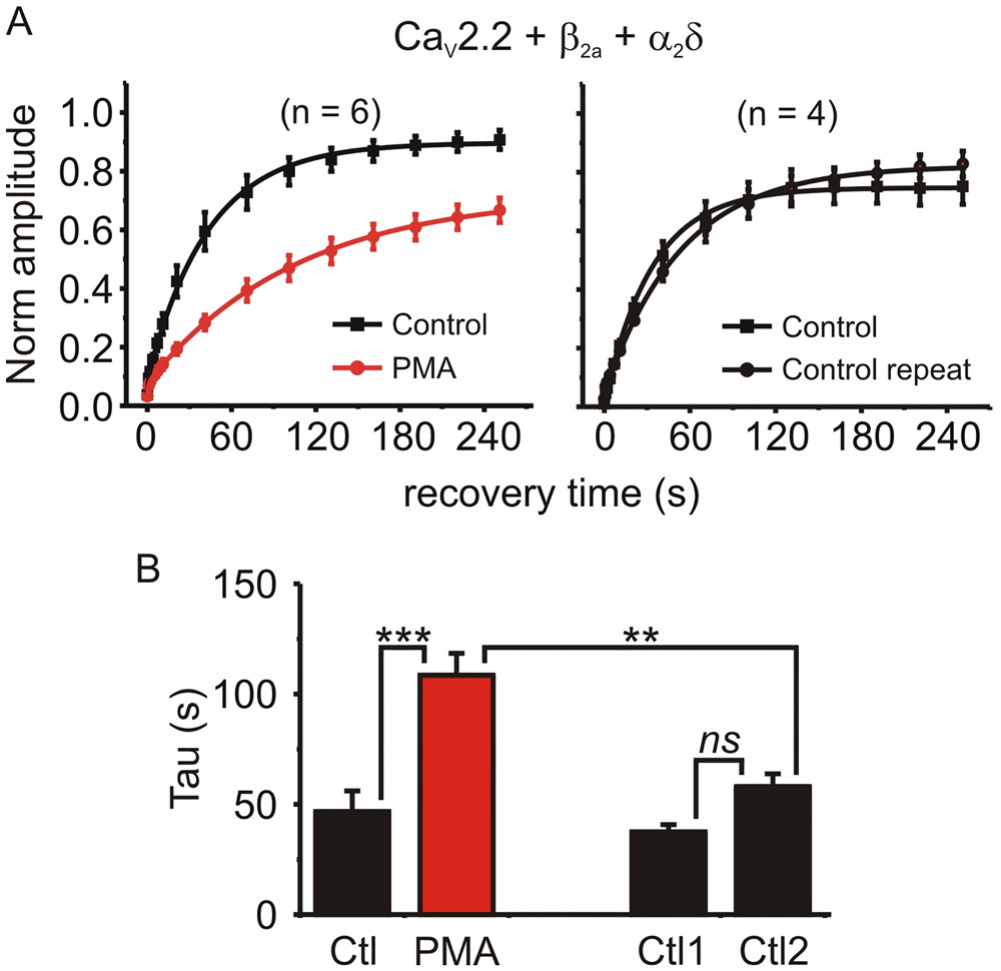
PMA significantly prolonged recovery from inactivation in β_2a_ containing channels. **(A)** HEK293 cells were transfected with Ca_V_2.2, α_2_δ, and β_2a_ subunits. Inactivation was produced by a 10s prepulse and recovery tracked as in Fig 1F. This was done twice in each cell: in some cells PMA was applied 5-min before the second stimulation protocol (left panel), while other cells were used as time-matched controls (i.e. no PMA was applied; right panel). Solid lines show exponential fits to the mean data (left panel: control A = 0.82, t = 40.4 s; PMA A = 0.65, t = 101 s, comparison of fits F = 24.2 p < 0.0001: right panel control A = 0.74, t = 36.9 s; control repeat A = 0.78, t = 55.8 s, comparison of fits F = 2.28 p = 0.1). **(B)** The time constant of the exponential fit to each individual cell was calculated and the means plotted. PMA significantly prolonged recovery from inactivation (* p < 0.05, ** p < 0.01, *ns* = “not significant” using ANOVA followed by Bonferroni’s multiple comparison test).

PMA significantly prolonged the recovery time constant in β_2a_ containing channels from 46.7 ± 9.4 s to 108.6 ± 9.9 s (n = 6, p = 0.00001, paired t-test) (Fig 3). We also tested the closely related Ca_V_2.1 (P/Q-type) channel (Ca_V_2.1, β_2a_ and α_2_δ). Recovery from inactivation in Ca_V_2.1 channels was faster than in Ca_V_2.2 channels, but was still significantly prolonged by PMA from 14.8 ± 3.2 s to 35.5 ± 9.7 s (n = 5, p = 0.04, paired t-test). To compare the effects of PMA across these channels with different subunit combinations that had inherently different rates of recovery, we calculated the change in recovery time constant (i.e. tau in the presence of PMA / tau before application of PMA). This tau ratio showed that PMA prolonged recovery from inactivation to a similar extent in all cases: the tau ratio was 2.66 ± 0.35 for Ca_V_2.2 + β_2a_ (n = 6), 3.09 ± 0.3 for Ca_V_2.2 + β_1b_ (n = 9), and 2.38 ± 0.38 for Ca_V_2.1 + β_2a_ (n = 5) (p = 0.34; F = 1.15, One-way ANOVA).

### PMA prolonged recovery from inactivation following trains of brief stimuli applied at physiologically relevant frequencies

**Fig 4:**
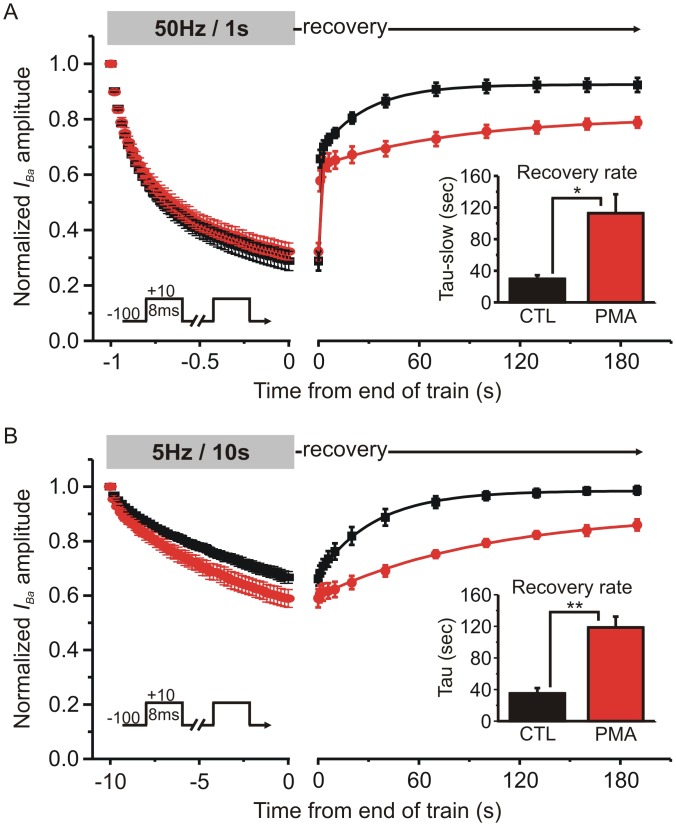
Recovery from “slow” inactivation following trains of brief stimuli was prolonged by PMA. **(A)** HEK293 cells expressing Ca_V_2.2, β_1b_ and α_2_δ were stimulated with a train of brief (8ms) step depolarizations (50Hz for 1s) and recovery from inactivation was tracked at various time points as indicated. *I*_*Ba*_ amplitude was normalized to the first pulse of the stimulus train. The left panel shows the current decline (inactivation) during the stimulus train and the right panel shows the time-course of recovery following the train (note the change in scale on the X axis). Solid lines show double exponential fits to the mean recovery data (control: Y_0_ = 0.27, A_1_ = 0.39, A_2_ = 0.27, t_1_ = 0.38 s, t_2_ = 24.8 s: PMA Y_0_ = 0.31, A_1_ = 0.33, A_2_ = 0.16, t_1_ = 0.56 s, t_2_ = 68.8 s, comparison of fits F = 8.98 p = 0.001). The inset bar graph shows the mean time constant for the slower component of recovery calculated from fits to the individual cells (* p = 0.045 paired t-test, n = 4). **(B)** The same layout as panel A, except that the cells were stimulated using a 5Hz train lasting 10 s. Solid lines show an exponential fit to the mean recovery data (control: Y_0_ = 0.67, A = 0.30, t = 32.4 s; PMA Y_0_ = 0.60, A = 0.31, t = 106.8 s, comparison of fits F = 79.25 p < 0.0001). The inset bar graph shows the mean time constant for recovery calculated from fits to the individual cells (** p = 0.0042 paired t-test, n = 6).

To more closely mimic physiologically relevant electrical activity, we stimulated cells with trains of brief depolarizations applied at 50Hz for 1s, or 5Hz for 10s (Fig 4). The extent of inactivation at the end of the 50Hz train was not altered by PMA (73% ± 3.6% in control conditions Vs. 69 ± 3.6% in PMA; n = 4; p = 0.21, paired t test) (Fig 4A). The recovery was biphasic, and PMA slightly reduced the magnitude of the initial fast component: in control conditions 52 ± 3% of the inactivation was recovered by 1s (the first recovery time point) and this was reduced to 40 ± 4% by PMA (n = 4, p = 0.021, paired t-test). PMA significantly prolonged the time constant of the slow recovery phase from 29.7 ± 4.6 s in control conditions to 112.9 ± 23.9 s in the presence of PMA (n = 4; p = 0.045 paired t-test). As expected, the 5Hz train produced less inactivation than the 50Hz train (Fig 4B). The extent of inactivation at the end of the 5Hz train was significantly increased by PMA (33 ± 2% Vs. 41 ± 3%, n = 6; p = 0.015, paired t-test). Recovery following the 5 Hz train was well fit with a single exponential and lacked the initial fast component seen after the 50 Hz train. The time constant of recovery was significantly slowed from 35.1 ± 6.7 s in control conditions to 118.5 ± 13.8 s in the presence of PMA (n = 6, p = 0.0042, paired t-test). These data show that PMA prolonged recovery from “slow” inactivation following physiologically relevant stimulus trains as well as following sustained step depolarization.

### Probing the involvement of PKC on recovery from “slow” inactivation

**Fig 5:**
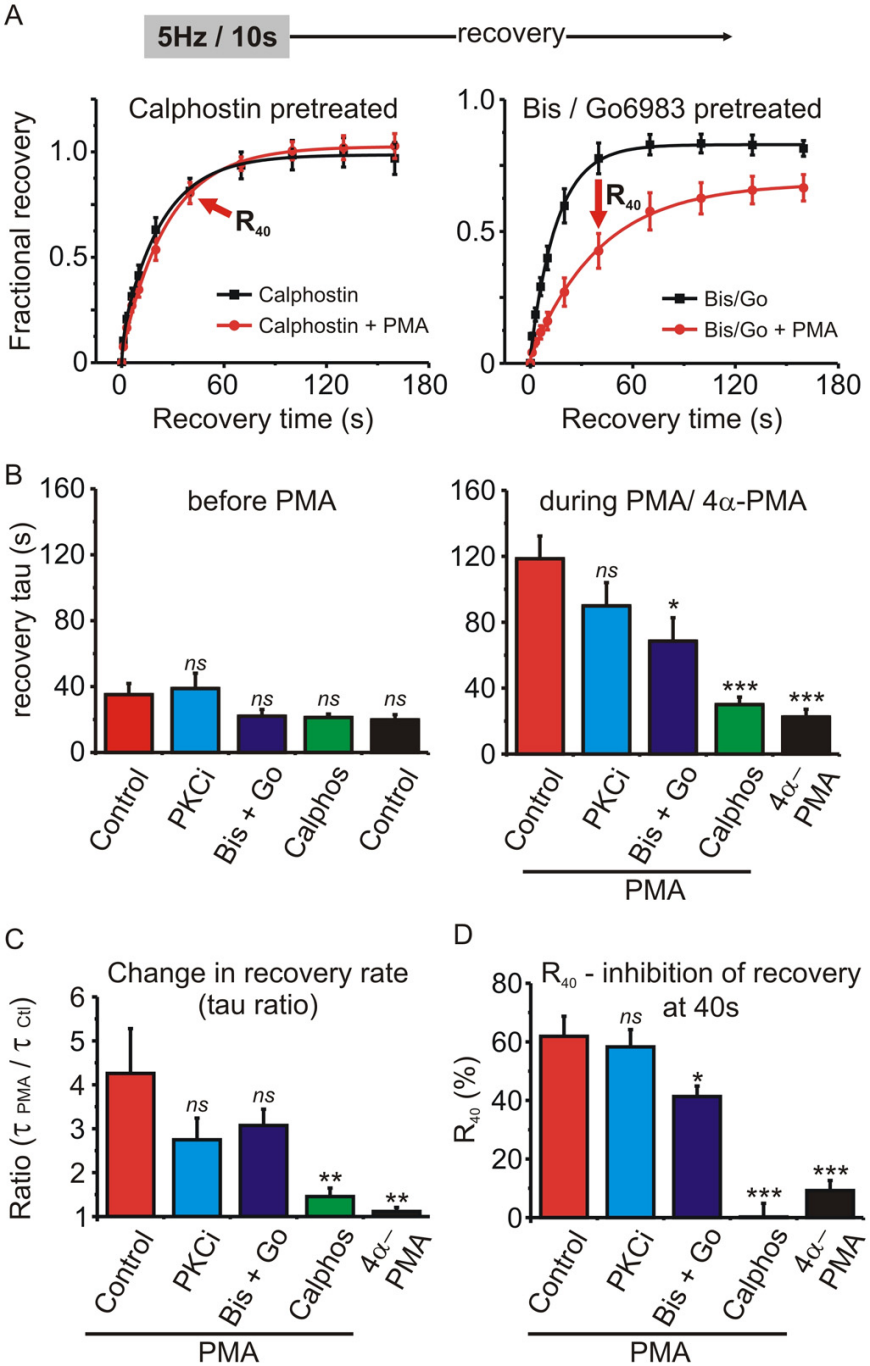
Testing the involvement of PKC in the prolonged recovery from inactivation produced by PMA. **(A)** Cells were pretreated with calphostin C (200 nM) or a mixture of bisindolylmaleimide-1 (Bis; 500 nM) + Go6983 (100 nM). Inactivation was produced by a stimulus train (5Hz for 10s as in Fig 4) and the fractional recovery from inactivation determined first in the absence and then in the presence of PMA. Solid lines show an exponential fit to the mean data (left panel: calphostin A = 0.95, t = 17.2 s; calphostin + PMA A = 1.0, t = 23.7 s, comparison of fits F = 3.62 p = 0.06: right panel: Bis/Go A = 0.80, t = 15.6 s; Bis/Go + PMA A = 0.67 t = 40.7 s, comparison of fits F = 25.3 p < 0.0001). The arrows labeled “R_40_“denote the 40s recovery time point. **(B)** Plots the mean recovery time constant determined from an exponential fit in each cell before (left panel) and during (right panel) application of PMA, or the inactive control 4α-PMA. “Control” = no pretreatment (n = 6); “PKCi” = cells recorded with intracellular application of a pseudosubstrate peptide inhibitor of PKC (2 μM PKC(19–36) (n = 6); “Bis + Go” = cells pretreated with bisindolylmaleimide-1 (500nM) + Go6983 (100nM) (n = 6); “Calphos” = cells pretreated with calphostin C (200 nM) (n = 7). Also shown is data for cells treated with 4α-PMA (n = 4). Pretreatment with the various drugs did not significantly alter the recovery time constant before application of PMA (left panel). The effect of PMA was significantly reduced by pretreatment with Bis + Go or calphostin C (right panel) (*ns*, not significantly different, * p < 0.05, *** P < 0.001 compared to the control PMA cells (red bar) determined using one-way ANOVA and Dunnett’s post-test). (**C**) To quantify the change in recovery rate produced by PMA, a ratio of the recovery time constants was calculated in each cell (tau in the presence of PMA / tau before application of PMA). Statistical significance compared to the control PMA cells (red bar) was determined using one-way ANOVA and Dunnett’s post-test (ns, not significantly different, ** P < 0.01). **(D)** Another index to compare the various drug treatments is the percent inhibition of recovery at the 40 s time point (R_40_—see panel A and results section for more detail). Statistical significance compared to the control PMA cells (red bar) was determined using one-way ANOVA and Dunnett’s post-test (ns, not significantly different, * p < 0.05, *** P < 0.001).

4α-PMA is an inactive control for PMA that does not activate PKC. We found that 4α-PMA did not significantly alter recovery from inactivation following a 5Hz/10s stimulus train in HEK cells expressing Ca_V_2.2, β_1b_ and α_2_δ, (tau control = 19.8 ± 3.1 s Vs tau 4α-phorbol = 22.5 ± 4.7 s; n = 4; p = 0.28, paired t-test). This is consistent with the involvement of PKC, so we tested several PKC inhibitors to see if they blocked the effect of PMA on recovery from inactivation. Cells were pre-incubated for 20–30 minutes with the inhibitors and then stimulated first in the presence of the inhibitor alone and then in the presence of the inhibitor + PMA. The inhibitors used were PKC(19–36), a pseudosubstrate peptide inhibitor of PKC that was added to the intracellular patch-pipette solution (2 μM); a combination of bisindolylmaleimide-1 (500nM) + Go6983 (100nM); calphostin C (200 nM). Recovery from inactivation following a 5Hz/10s train was fit with an exponential to determine the recovery time constant (Fig 5A and 5B). None of the drug pretreatments significantly altered the baseline recovery rate prior to application of PMA (Fig 5B) (p = 0.09, F = 2.28; one-way ANOVA followed by Dunnett’s post-test). However, the recovery time constant in the presence of PMA was significantly reduced by pretreatment with bisindolylmaleimide-1 + Go6983 or calphostin C (p < 0.0001, F = 11.64; one-way ANOVA followed by Dunnett’s post-test for multiple pairwise comparisons) (Fig 5B). To compare the various treatment groups, the change in recovery time constant was expressed as a ratio (tau in the presence of PMA / tau before PMA) (Fig 5C). The slowing of recovery produced by PMA (tau ratio) was not seen with the control 4α-PMA, was abolished by pretreatment with calphostin C, and partially, but not significantly reduced in cells pretreated with PKC(19–36), or bisindolylmaleimide-I + Go6983 (p = 0.005, F = 4.885, one-way ANOVA, followed by Dunnett’s post-test). It has been reported previously that the extent, but not the rate, of Ca_V_2.2 recovery from inactivation can be modulated by alternative splicing [37]. Therefore, we calculated the percent change in recovery produced by PMA at the 40 s time point (R_40_) (Fig 4A and 4C). In control cells (before application of PMA) the fractional recovery at this time was 0.67 ± 0.07, and in the presence of PMA this was reduced to 0.23 ±0.02 (n = 6, p = 0.0026, paired t-test) resulting in a calculated R_40_ of 61 ± 6.9%. We calculated R_40_ for the various treatment groups (different PKC inhibitors) and found it was significantly reduced by bisindolylmaleimeide-1 + Go6983 and abolished by calphostin C (Fig 5D) (p = 0.0001, F = 29.3, one-way ANOVA followed by Dunnett’s post-test). Thus, our data show that the effect of PMA on recovery from inactivation was abolished by calphostin C and partially blocked by bisindolylmaleimeide-1 + Go6983.

**Fig 6:**
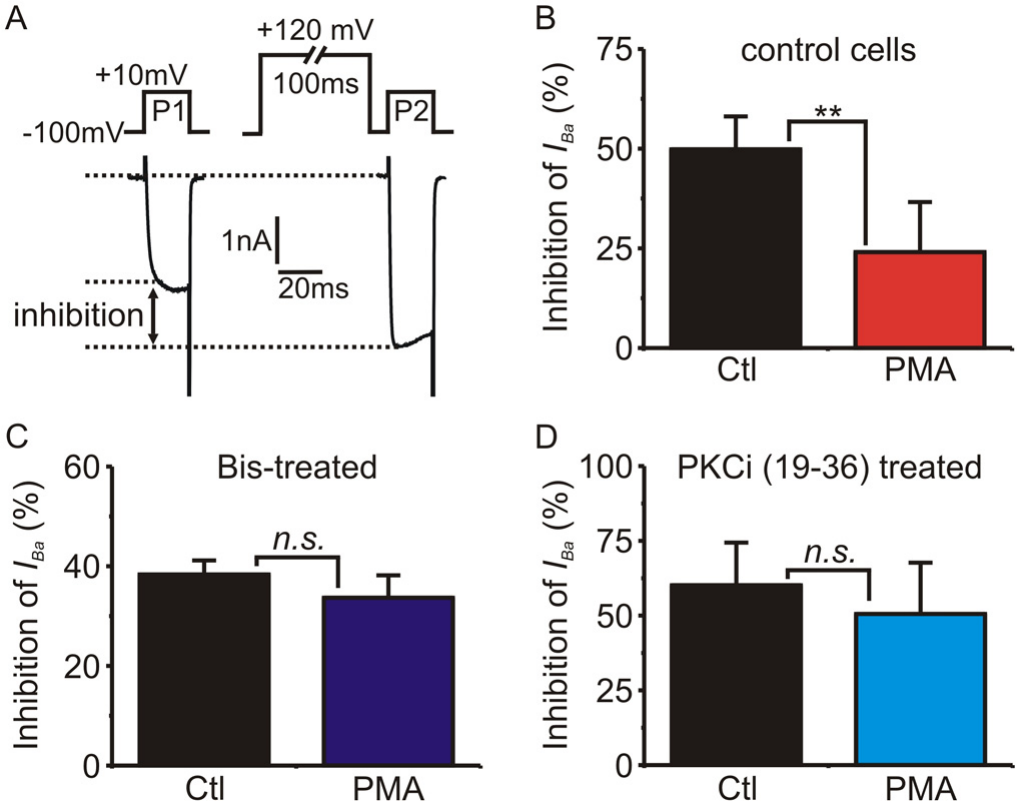
Bisindolylmaleimide-1 and PKC(19–36) effectively block the effects of PKC / phorbol ester on Gβγ-mediated inhibition of *I*_*Ba*_. G1A1 cells (that stably express Ca_V_2.2, β_1b_ and α_2_δ) were transiently transfected with G-protein β_1_ and γ_2_ subunits (Gβγ). **(A)** Representative traces showing use of a depolarizing prepulse protocol to quantify the tonic inhibition of *I*_*Ba*_ produced by Gβγ. **(B)** The magnitude of Gβγ-mediated inhibition was determined using the prepulse protocol (panel A) first in the absence and then in the presence of PMA (200 nM). PMA significantly diminished the Gβγ-mediated inhibition (p = 0.0015, n = 5, paired t-test). **(C)** Preincubation of cells with bisindolylmaleimide-1 (Bis; 500 nM) blocked the ability of PMA to reduce Gβγ-mediated inhibition (p = 0.75, n = 5, paired t-test). **(C)** PKC(19–36) in the patch pipette solution blocked the ability of PMA to reduce Gβγ-mediated inhibition (p = 0.09, n = 3, paired t-test).

Given the incomplete block of PMA by bisindolylmaleimide-I and especially PKC(19–36) (see above and Fig 5), we wanted to test their ability to antagonize PKC under our recording conditions. To do so, we investigated the inhibition of Ca_V_2.2 channels by G protein βγ subunits (Gβγ) [1, 2]. Gβγ inhibits *I*_*Ba*_ by binding directly to the Ca_V_2.2 subunit, and activation of PKC by phorbol ester has been shown to partially reverse this inhibition [38–43]. We transiently transfected G1A1 cells with GFP-tagged Gβγ subunits (Fig 6) which results in tonic inhibition of *I*_*Ba*_. A trademark characteristic of Gβγ-mediated inhibition is that it can be reversed by strong membrane depolarization due to transient voltage-dependent dissociation of Gβγ from the channel [1]. Thus, the extent of Gβγ-mediated inhibition can be revealed using a prepulse facilitation protocol as shown in Fig 6A. Treating cells with PMA for 5-minutes significantly reduced the extent of Gβγ-mediated inhibition (Fig 6B). Pretreating cells with bisindolylmaleimide-I (Fig 6C) or PKC(19–36) (Fig 6D) blocked the ability of PMA to reduce Gβγ-mediated inhibition. Thus the drugs were effective under our recording conditions, suggesting the partial inhibition of PMA’s actions on slow inactivation is not simply due to inactive drugs or other artefacts.

PMA can also activate some additional signaling pathways, for example by binding to the C1-domain of RasGRP (Ras Guanine nucleotide Releasing Protein) which in turn leads to activation of the monomeric G protein Ras [44, 45]. To investigate a potential role for Ras signaling we transfected G1A1 cells with Ras S17N (a dominant negative mutant) or Ras G12V (a constitutively active mutant) and compared recovery to control cells transfected with GFP alone. Neither of the Ras mutants altered the baseline recovery rate before application of PMA, nor did they alter the slowing of recovery produced by PMA. The change in recovery rate (tau ratio) produced by PMA was 3.09 ± 0.3 in GFP controls (n = 9), 2.42 ± 0.34 in S17N (n = 7) and 2.55 ± 0.26 in G12V (n = 7) (p = 0.25, F = 1.483, one-way ANOVA). Similarly, R_40_ (% inhibition of recovery at 40s by PMA) was not significantly altered by the Ras mutants: 36 ± 4% in controls, 27 ± 7% in S17N and 31 ± 6% in G12V (p = 0.53, F = 0.664, one-way ANOVA). Phorbol esters / PKC have also been reported to modulate surface expression of some ion channels and transporters through dynamin dependent endocytosis [46–49] and Ca_V_2 channels are known to associate with endocytic complexes and are subject to internalization by various stimuli [50–53]. However, when a dynamin inhibitory peptide (50 μM) was included in the patch-pipette solution, PMA still significantly slowed the recovery time constant in HEK cells expressing Ca_V_2.2, β_1b_ and α_2_δ, from 28.9 ± 3 s to 65.5 ± 14.1 s (n = 5; p = 0.033 paired t-test) suggesting endocytosis is not playing a significant role under our recording conditions.

### The endogenous *I*_*Ca*_ in neuroendocrine chromaffin cells is regulated in a similar manner by PMA

It is possible that channels in heterologous expression systems are regulated in a different manner than would be the case in a native cellular environment. Therefore, we investigated the ability of PMA to slow recovery of *I*_*Ca*_ from inactivation in adrenal chromaffin cells, an important neuroendocrine component of the sympathetic nervous system [54]. In bovine chromaffin cells Ca_V_2 channels account for ~85–90% of the whole cell calcium current in a roughly 1:1 ratio of N-type and P/Q-type *I*_*Ca*_, with the remainder due primarily to a small (~10%) Ca_V_1 (L-type) component [36, 55, 56]. As before, we included 10mM BAPTA in the patch pipette solution which effectively blocked calcium dependent inactivation of *I*_*Ca*_ to enable investigation of voltage-dependent inactivation [22, 57]. However, we used Ca^2+^ as the extracellular divalent charge carrier because Ba^2+^ depolarizes the surrounding cells in the recording chamber leading to exocytosis of catecholamines, ATP, and opioids that can act in a paracrine manner to modulate the *I*_*Ca*_ in the cell being recorded from [36].

**Fig 7:**
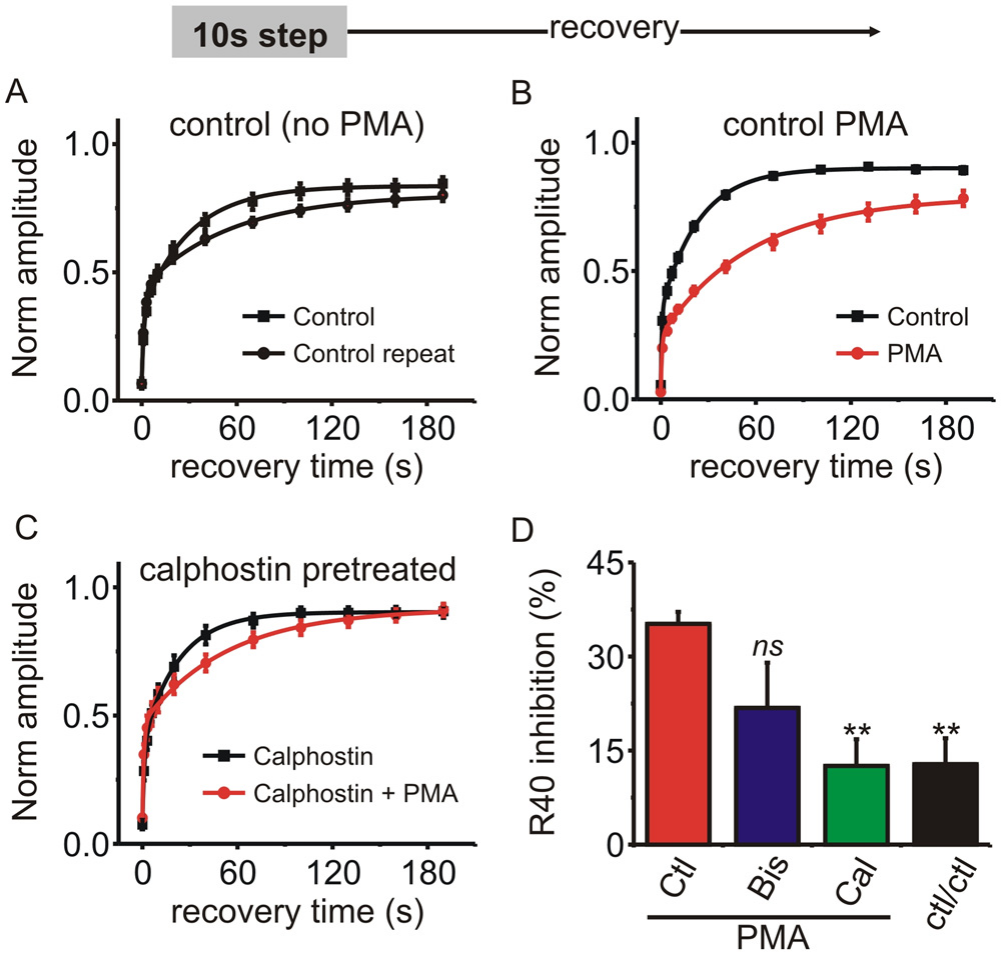
PMA prolonged recovery of *I*_*Ca*_ from inactivation in adrenal chromaffin cells. **(A–C)** Chromaffin cells were stimulated with a 10s step depolarization and recovery from inactivation tracked at the indicated time points. *I*_*Ca*_ amplitude (normalized to that before the 10 step depolarization) is plotted for each of the recovery time points and the mean data fit with a double exponential (solid lines). A) Control cells with no PMA treatment (n = 11) (fit parameters: control Y_0_ = 0.07, A_1_ = 0.29, A_2_ = 0.49, t_1_ = 1.5 s, t_2_ = 32.1 s; control repeat Y_0_ = 0.07, A_1_ = 0.36, A_2_ = 0.37, t_1_ = 1.5 s, t_2_ = 52.6 s: fit comparison F = 1.66 p = 0.21). B) Control cells with PMA (n = 6) (fit parameters: control Y_0_ = 0.05, A_1_ = 0.28, A_2_ = 0.56, t_1_ = 0.7 s, t_2_ = 23.5 s; PMA Y_0_ = 0.03, A_1_ = 0.24, A_2_ = 0.55, t_1_ = 1.1 s, t_2_ = 69.6 s, comparison of fits F = 35.2 p < 0.0001). C)Cells pretreated with calphostin C (n = 6) (fit parameters: calphostin Y_0_ = 0.08, A_1_ = 0.31, A_2_ = 0.52, t_1_ = 1.2 s, t_2_ = 22.6 s; calphostin + PMA Y_0_ = 0.10, A_1_ = 0.36, A_2_ = 0.45, t_1_ = 1.0 s, t_2_ = 52.7 s, comparison of fits F = 1.73 p = 0.19). **(D)** The percent inhibition of recovery at the 40 s time point was calculated (R_40_—see results section for more detail). The bar graph shows R_40_ produced by PMA in control cells (PMA; n = 6), cells pretreated with bisindolylmaleimide-1 (Bis; n = 4), and cells pretreated with calphostin C (Cal; n = 6). The R_40_ for control cells stimulated twice in the absence of PMA is also shown (ctl/ctl; n = 11). Statistical significance compared to the control PMA cells (red bar) was determined using one-way ANOVA and Dunnett’s post-test (ns, not significantly different, ** p < 0.01).

“Fast” voltage-dependent inactivation is minimal in chromaffin cells, possibly due to expression of the β_2a_ subunit [58, 59]. However, the 10s step depolarization paradigm produced robust inactivation of *I*_*Ca*_ both in control conditions (95 ± 1%) and in the presence of PMA (97 ± 0.5%, n = 7; p = 0.132, paired t-test). Recovery from inactivation exhibited at least two kinetic components (Fig 7). PMA clearly prolonged recovery from inactivation, with a particularly prominent effect on the slower time constant which was increased from 26.6 ± 1.4 s to 90.9 ± 8.8 s (n = 7, p = 0.0002, paired t-test). The R_40_ (% change in recovery at 40s) showed that PMA inhibited recovery by 35.5 ± 1.6% (n = 7). Pretreating cells with bisindolylmaleimide-1 modestly reduced R_40_ to 21.6 ± 7% (n = 5) although this was not statistically significant, and calphostin C significantly reduced R_40_ to 12.8 ± 4% (n = 6; p < 0.01 one-way ANOVA followed by Dunnetts post-test) (Fig 7D). These data mirror those obtained in recombinant channels and confirmed that PMA prolongs recovery from “slow” inactivation for endogenously expressed *I*_*Ca*_ in neuroendocrine cells.

### GDP-β-S reduces the slowing of recovery produced by PMA

**Fig 8:**
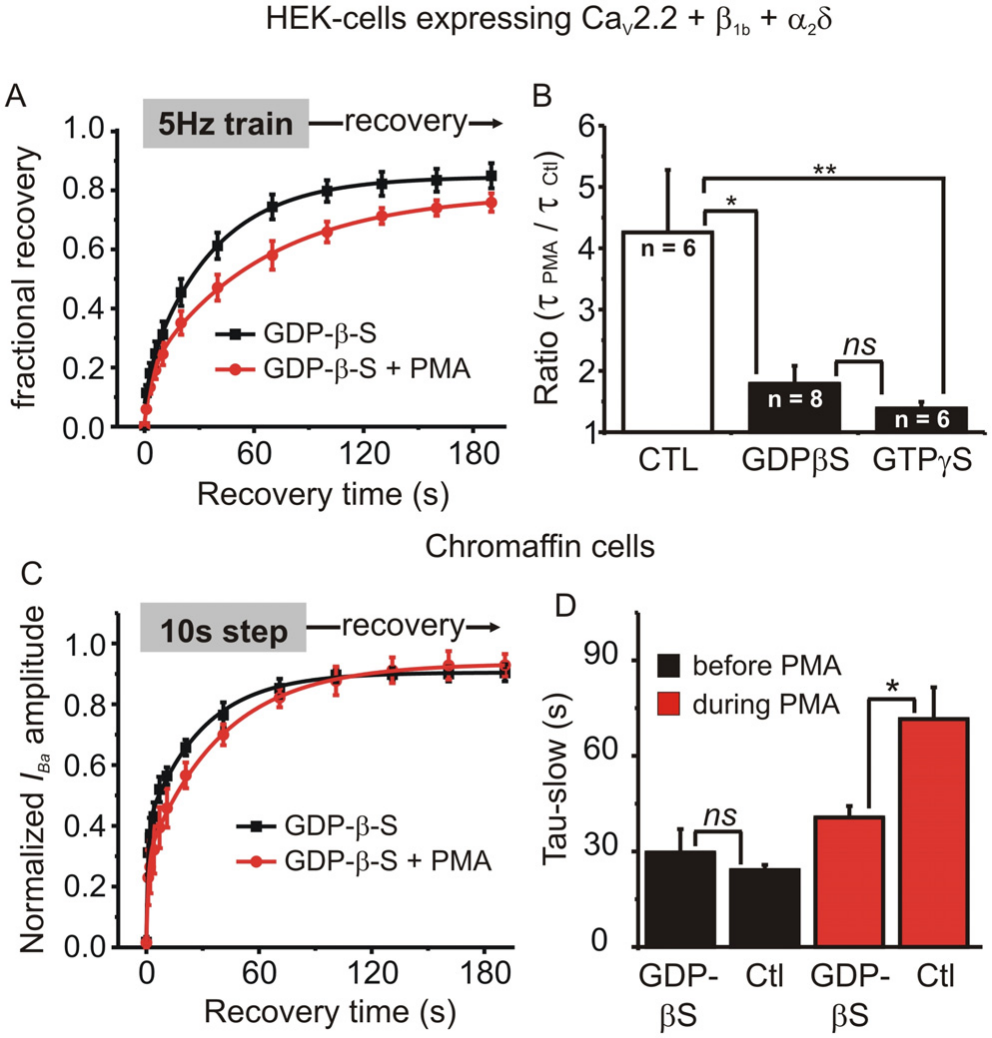
Intracellular application of GDP-β-S or GTP-γ-S reduced the effect of PMA on recovery from inactivation. **(A)** HEK293 cells expressing Ca_V_2.2, β_1b_ and α_2_δ were recorded with patch pipette solution containing 0.5mM GDP-β-S and stimulated with a 5Hz/10s train. Fractional recovery from inactivation is plotted over time and the solid lines show a double exponential fit to the mean data (fit parameters: GDP-β-S A_1_ = 0.13, A_2_ = 0.71, t_1_ = 0.9 s, t_2_ = 35.0 s; GDP-β-S + PMA A_1_ = 0.18, A_2_ = 0.61, t_1_ = 3.6 s, t_2_ = 62.7 s, comparison of fits F = 12.9 p < 0.0001). **(B)** The change in recovery rate produced by PMA (tau in the presence of PMA / tau before application of PMA) for control cells, cells recorded with GDP-β-S in the patch pipette solution, and cells recorded with GTP-γ-S in the patch pipette solution (ns, not significant, * p < 0.05, ** p < 0.01; ANOVA with Bonferroni’s post-test for multiple pairwise comparisons). **(C)** Endogenous *I*_*Ca*_ was recorded from adrenal chromaffin cells with GDP-β-S in the patch pipette solution. Chromaffin cells were stimulated with a 10s step depolarization and recovery from inactivation was tracked at the indicated time points before and during application of PMA. The mean recovery data was fit with a double exponential (fit parameters: GDP-β-S Y_0_ = 0.02, A_1_ = 0.36, A_2_ = 0.52, t_1_ = 0.8 s, t_2_ = 28.1 s; GDP-β-S + PMA Y_0_ = 0.02, A_1_ = 0.25, A_2_ = 0.66, t_1_ = 0.8 s, t_2_ = 37.4 s, comparison of fits F = 1.65 p = 0.2). **(D)** Mean (± sem) data are shown for the slower of the two time constants from control cells (Ctl, n = 7) and cells recorded with GDP-β-S (n = 4). GDP-β-S had no effect on recovery rate in the absence of PMA (black bars) but significantly reduced the slowing of recovery produced by PMA (red bars) (ns, not significant, * p < 0.05; ANOVA with Bonferroni’s post-test for multiple pairwise comparisons).

To investigate any potential involvement of G protein signaling in the effects of PMA we replaced GTP with GDP-β-S (0.5 mM) in the intracellular patch pipette solution to prevent activation of heterotrimeric and monomeric G proteins (Fig 8). First we used this solution to record from HEK cells expressing Ca_V_2.2, β1b and α2δ subunits stimulated with a 5Hz/10s train. In the absence of PMA, GDP-β-S increased the extent of inactivation at the end of the 5Hz train compared to control cells (50 ± 4%, n = 8 Vs 34 ± 2%, n = 6; p = 0.011, unpaired t-test). The rate of recovery from inactivation under control conditions (before application of PMA) was not altered by GDP-β-S, but the slowing of recovery produced by PMA was significantly reduced, as reflected in the tau ratio (Fig 8A and 8B). We repeated a similar experiment but with GTP-γ-S added to the patch-pipette solution. Again, the prolonged recovery from inactivation produced by PMA was blocked in these cells (Fig 8B). Similarly, in adrenal chromaffin cells GDP-β-S had no effect on recovery from inactivation under control conditions, but significantly reduced the slowing of recovery produced by PMA (Fig 8C and 8D). Together these data suggest that G protein signaling pathways can influence the kinetics of recovery from inactivation, opposing the actions of PMA.

## Discussion

### PMA prolongs recovery from “slow” inactivation

Voltage-dependent inactivation of Ca_V_2.2 plays an important role in controlling the temporal response of the channels to different electrical stimuli. “Fast” inactivation is relatively well-understood and thought to involve pore occlusion by the cytoplasmic I-II linker [11, 18, 19]. As with other voltage-gated channels, there is an additional mode of inactivation with slower onset and recovery kinetics. This “slow” inactivation is molecularly distinct but poorly understood. It has been investigated somewhat more in sodium and potassium channels where proposed mechanism(s) involve altered movement of the voltage-sensor and/or constriction of the channel pore [26–29]. “Slow” inactivation of sodium channels is modulated by second messengers including PKA and PKC, which in turn controls neuronal excitability [30, 31, 60, 61]. It has been reported that co-expression with syntaxin can promote “slow” inactivation of Ca_V_2 channels [25] but little else was known about the how it might be controlled. Therefore, we set out to investigate “slow” inactivation of Ca_V_2.2 channels using both recombinant channels expressed in HEK293 cells, and endogenous channels in adrenal chromaffin cells. In both these cell types the Ca_V_2 channels undergo Ca^2+^-dependent inactivation which confounds investigation of voltage-dependent inactivation. Therefore, we included 10mM BAPTA in the patch pipette solution and, for HEK293 cells, we also used Ba^2+^ rather than Ca^2+^as the charge carrier to prevent Ca^2+^-dependent inactivation.

In the literature, the effects of PMA/PKC on Ca_V_2.2 channels are mixed/complex. In some cases there is a pronounced increase in current amplitude, at least in part due to reversal of tonic Gβγ-mediated inhibition [38–43, 62]. PKC can also inhibit or exert bidirectional modulation of *I*_*Ca*_, perhaps through distinct kinase isoforms and phosphorylation sites, and its effects might be altered by the Ca_V_β subunit [63–67]. We found there was little effect of PMA on *I*_*Ba*_ amplitude (Fig 1). This might reflect a lack of tonic inhibition by Gβγ under our conditions, or could be due to the strong Ca^2+^ buffering (10mM BAPTA) which might preclude activation of classic (Ca^2+^-dependent) PKC isoforms. We also found no effect of PMA on the I-V curve, inactivation curve, or “fast” inactivation during a step depolarization (Fig 1). During a step depolarization the onset of “fast” inactivation masks that of “slow” inactivation. However, tracking the recovery from inactivation provides a convenient solution: recovery from “fast” inactivation is similarly fast, whereas recovery from “slow” inactivation is slow. Thus we focused on recovery from inactivation following different stimulus protocols, and how this was modulated by the phorbol ester PMA. Recovery following a 10s step depolarization was biphasic with the slower component accounting for >80% of the total. As expected for recovery from voltage-dependent inactivation, the kinetics of this slow component showed no correlation with amount of barium entry (Fig 2B) and were accelerated at more hyperpolarized potentials (Fig 2C). When cells were stimulated with a 1s step depolarization recovery was faster than following a 10s step consistent with the prediction that fewer channels enter the “slow” inactivated state during a shorter duration stimulus (Fig 1E and 1F). We show that PMA dramatically prolonged recovery from “slow” inactivation with little impact on recovery from “fast” inactivation. We found a similar effect when using a train of brief stimuli to more closely resemble physiological patterns of electrical activity (Fig 4). Importantly, we show that PMA also prolonged recovery of Ca_V_2 channels from “slow” inactivation in adrenal chromaffin cells, an important neuroendocrine component of the sympathetic nervous system (Fig 7). Thus, this phenomenon is apparent in a native cellular context and is not an artefact of the heterologous expression system. Taken together, we interpret our data as reflecting the activity-dependent entry of Ca_V_2.2 channels into a “slow” inactivated state during sustained periods of stimulation, and that PMA prolongs recovery from this state.

Although we did not systematically compare the effects of different β subunits, we did investigate the β_2a_ subunit because it dramatically reduces “fast” inactivation. Recovery from inactivation took significantly longer with β_2a_ than in cells with β_1b_. It was previously reported that the β subunit had no effect on recovery from “slow” inactivation in Ca_V_2.1 channels, although it did indirectly modulate the onset kinetics via altered fast inactivation [24]. It is possible that the β subunit has a different impact on “slow” inactivation of Ca_V_2.2 and CaV2.1 channels, but this will need to be confirmed by direct comparison in future investigations. The focus of our study was the impact of PMA on “slow” inactivation and we found that, despite the different baseline recovery rates, PMA produced a similar ~2.5–3 fold increase in the recovery time constant for Ca_V_2.2 channels containing either the β_2a_ or β_1b_ subunits. We also found that the rate of recovery Ca_V_2.1 channels (with β_2a_) following a 10s step was faster than for the Ca_V_2.2 channels, but still prolonged by treatment with PMA. Thus, PMA prolongs recovery from “slow” inactivation in both Ca_V_2 channel types, and it will be interesting to determine if this extends to the Ca_V_1 or Ca_V_3 Ca^2+^channels.

### Does PKC underlie the slowed recovery from inactivation produced by PMA?

Recovery from inactivation was prolonged by PMA but not by 4α-PMA, a control analogue that does not activate PKC. Also consistent with the involvement of PKC, pretreating cells with calphostin C prevented the slowing of recovery produced by PMA (Fig 5). However, a combination of bisindolylmaleimide-1 + Go6983 only partially reduced the effect of PMA while PKC(19–36) had little effect. Similarly, in adrenal chromaffin cells calphostin C blocked the action of PMA while bisindolylmaleimide-1 only had a partial, statistically non-significant effect (Fig 7D). Both PKC(19–36) and bisindolylmaleimide-1 were able to effectively antagonize the ability of PMA to reverse Gβγ-mediated inhibition of Ca_V_2.2 channels (Fig 6). So why were these drugs less effective at antagonizing the effect of PMA on “slow” inactivation? One clue might come from the mechanism of action of the different antagonists: calphostin C targets the regulatory C1-domain of PKC whereas the other antagonists target the catalytic domain. It has been shown that A-kinase anchoring protein-79 (AKAP-79) scaffolds a signaling complex between PKC and KCNQ potassium channels [68]. Moreover, when PKC is in this complex it is protected from antagonists that target the ATP binding catalytic domain, but still inhibited by calphostin C [68]. Perhaps a similar situation exists for the Ca_V_2 channels, which would explain the differential sensitivity to the PKC antagonists.

The effects of phorbol esters / PMA are not always recapitulated by stimulating endogenous pathways that activate PKC, such as Gq-coupled GPCRs. One possible explanation is that PMA acts at least in part through a non-PKC signaling pathway. Phorbol esters can bind to the C1-domain of other proteins, for example RasGRPs which activate the monomeric G protein Ras [44, 45]. However, our data suggest this pathway is not involved, because overexpression of constitutively active or dominant negative Ras mutants had no effect on the ability of PMA to slow recovery from inactivation. Potential involvement of other C1-domain proteins will require further investigation. PMA has also been reported to promote removal of ion channels and transporters from the plasma membrane through dynamin-dependent endocytosis [46–49]. Although we cannot categorically rule out a role for channel trafficking, our data are not consistent with this playing a major role. First, a dynamin inhibitory peptide did not significantly change the effect of PMA on recovery from inactivation. Second, PMA had no effect on the amplitude of *I*_*Ba*_ prior to the stimulus train / 10s step, and the extent of inactivation was only modestly altered. Recovery from fast inactivation was unaltered by PMA and recovery from slow inactivation was voltage-dependent (Fig 2C). None of these features are consistent with endocytic recycling of the channels playing a major role under our recording conditions.

Finally, our data also point to a role for G protein signaling in helping to control slow inactivation. Disrupting G protein signaling using intracellular GDP-β-S had no obvious effect on recovery from inactivation *per se*, but significantly reduced the ability of PMA to slow recovery (Fig 8B). Similarly, GTP-γ-S (which should activate rather than block G protein signaling) also reduced the effects of PMA. While it is possible that PMA recruits a second messenger pathway involving G protein signaling, it is also possible that G proteins or guanosine nucleosides exert a parallel, allosteric effect that is permissive for the actions of PMA. This possibility will require extensive future investigations.

## Conclusions

“Fast” voltage-dependent inactivation of Ca^2+^ channels is relatively well characterized and helps shape channel activity and short-term synaptic depression during brief trains of action potentials (tens-hundreds of milliseconds). Superimposed on this is the molecularly distinct, but poorly understood process of “slow” inactivation which develops and recovers over the course of seconds-to-minutes. The slow onset favors recruitment by sustained periods of electrical activity or membrane depolarization, while the slow recovery kinetics might confer a short-term “molecular memory” for this preceding cellular activity. In this study we report the novel finding that PMA dramatically prolongs recovery of Ca_V_2.2 channels from “slow” inactivation. This effect is likely mediated at least in part through PKC and might also involve G protein / guanosine nucleoside signaling. Regardless of the precise molecular details, this could provide a novel mechanism for dynamic, activity-dependent regulation of Ca^2+^channel availability, electrical excitability, and neurotransmission in the seconds-to-minutes timeframe.

## References

[1] Zamponi GW, Currie KP. Regulation of Ca(V)2 calcium channels by G protein coupled receptors. Biochim Biophys Acta. 2013;1828(7):1629–43. Epub 2012/10/16. 10.1016/j.bbamem.2012.10.004 23063655 PMC3556207

[2] Currie KP. G protein inhibition of CaV2 calcium channels. Channels (Austin, Tex. 2010;4(6):497–509. Epub 2010/12/15. 12871 [pii]. . 21150298 10.4161/chan.4.6.12871 PMC3052249

[3] Ben-Johny M, Yue DT. Calmodulin regulation (calmodulation) of voltage-gated calcium channels. J Gen Physiol. 2014;143(6):679–92. Epub 2014/05/28. 10.1085/jgp.201311153 ; PubMed Central PMCID: PMCPmc4035741. 24863929 PMC4035741

[4] Catterall WA, Leal K, Nanou E. Calcium channels and short-term synaptic plasticity. J Biol Chem. 2013;288(15):10742–9. Epub 2013/02/13. 10.1074/jbc.R112.411645 ; PubMed Central PMCID: PMCPmc3624454. 23400776 PMC3624454

[5] Catterall WA. Structure and regulation of voltage-gated Ca2+ channels. Annu Rev Cell Dev Biol. 2000;16:521–55. . 11031246 10.1146/annurev.cellbio.16.1.521

[6] Roberts-Crowley ML, Mitra-Ganguli T, Liu L, Rittenhouse AR. Regulation of voltage-gated Ca2+ channels by lipids. Cell Calcium. 2009;45(6):589–601. Epub 2009/05/08. S0143-4160(09)00060-8 [pii] 10.1016/j.ceca.2009.03.015 . 19419761 PMC2964877

[7] Christel C, Lee A. Ca2+-dependent modulation of voltage-gated Ca2+ channels. Biochim Biophys Acta. 2012;1820(8):1243–52. Epub 2012/01/10. 10.1016/j.bbagen.2011.12.012 ; PubMed Central PMCID: PMCPmc3345169. 22223119 PMC3345169

[8] Stephens GJ. G-protein-coupled-receptor-mediated presynaptic inhibition in the cerebellum. Trends Pharmacol Sci. 2009;30(8):421–30. Epub 2009/07/28. S0165-6147(09)00111-4 [pii] 10.1016/j.tips.2009.05.008 . 19632729

[9] Catterall WA, Few AP. Calcium channel regulation and presynaptic plasticity. Neuron. 2008;59(6):882–901. 10.1016/j.neuron.2008.09.005 18817729

[10] Cens T, Rousset M, Leyris JP, Fesquet P, Charnet P. Voltage- and calcium-dependent inactivation in high voltage-gated Ca(2+) channels. Prog Biophys Mol Biol. 2006;90(1–3):104–17. . 16038964 10.1016/j.pbiomolbio.2005.05.013

[11] Stotz SC, Jarvis SE, Zamponi GW. Functional roles of cytoplasmic loops and pore lining transmembrane helices in the voltage-dependent inactivation of HVA calcium channels. J Physiol. 2004;554(Pt 2):263–73. Epub 2003/06/20. jphysiol.2003.047068 [pii]. 12815185 10.1113/jphysiol.2003.047068 PMC1664770

[12] Hering S, Berjukow S, Sokolov S, Marksteiner R, Weiss RG, Kraus R, et al Molecular determinants of inactivation in voltage-gated Ca2+ channels. J Physiol. 2000;528 Pt 2:237–49. . 11034614 10.1111/j.1469-7793.2000.t01-1-00237.x PMC2270139

[13] DeMaria CD, Soong TW, Alseikhan BA, Alvania RS, Yue DT. Calmodulin bifurcates the local Ca2+ signal that modulates P/Q-type Ca2+ channels. Nature. 2001;411(6836):484–9. . 11373682 10.1038/35078091

[14] Lee A, Wong ST, Gallagher D, Li B, Storm DR, Scheuer T, et al Ca2+/calmodulin binds to and modulates P/Q-type calcium channels. Nature. 1999;399(6732):155–9. . 10335845 10.1038/20194

[15] Liang H, DeMaria CD, Erickson MG, Mori MX, Alseikhan BA, Yue DT. Unified mechanisms of Ca2+ regulation across the Ca2+ channel family. Neuron. 2003;39(6):951–60. . 12971895 10.1016/s0896-6273(03)00560-9

[16] Lee A, Zhou H, Scheuer T, Catterall WA. Molecular determinants of Ca(2+)/calmodulin-dependent regulation of Ca(v)2.1 channels. Proc Natl Acad Sci U S A. 2003;100(26):16059–64. . 14673106 10.1073/pnas.2237000100 PMC307692

[17] Kim EY, Rumpf CH, Fujiwara Y, Cooley ES, Van Petegem F, Minor DL Jr. Structures of CaV2 Ca2+/CaM-IQ domain complexes reveal binding modes that underlie calcium-dependent inactivation and facilitation. Structure (London, England: 1993). 2008;16(10):1455–67. Epub 2008/10/23. 10.1016/j.str.2008.07.010 ; PubMed Central PMCID: PMCPmc2701236. 18940602 PMC2701236

[18] Herlitze S, Hockerman GH, Scheuer T, Catterall WA. Molecular determinants of inactivation and G protein modulation in the intracellular loop connecting domains I and II of the calcium channel alpha1A subunit. Proc Natl Acad Sci U S A. 1997;94(4):1512–6. . 9037084 10.1073/pnas.94.4.1512 PMC19822

[19] Zhang JF, Ellinor PT, Aldrich RW, Tsien RW. Molecular determinants of voltage-dependent inactivation in calcium channels. Nature. 1994;372(6501):97–100. . 7969428 10.1038/372097a0

[20] Dolphin AC. Calcium channel auxiliary alpha2delta and beta subunits: trafficking and one step beyond. Nat Rev Neurosci. 2012;13(8):542–55. Epub 2012/07/19. 10.1038/nrn3311 nrn3311 [pii]. . 22805911

[21] Buraei Z, Yang J. Structure and function of the beta subunit of voltage-gated Ca(2)(+) channels. Biochim Biophys Acta. 2013;1828(7):1530–40. Epub 2012/09/18. 10.1016/j.bbamem.2012.08.028 ; PubMed Central PMCID: PMCPmc3587009. 22981275 PMC3587009

[22] McDavid S, Currie KP. G-proteins modulate cumulative inactivation of N-type (Cav2.2) calcium channels. J Neurosci. 2006;26(51):13373–83. Epub 2006/12/22. 26/51/13373 [pii] . 17182788 10.1523/JNEUROSCI.3332-06.2006 PMC6675003

[23] Jones SW, Marks TN. Calcium currents in bullfrog sympathetic neurons. II. Inactivation. J Gen Physiol. 1989;94(1):169–82. . 2553857 10.1085/jgp.94.1.169 PMC2228927

[24] Sokolov S, Weiss RG, Timin EN, Hering S. Modulation of slow inactivation in class A Ca2+ channels by beta-subunits. J Physiol. 2000;527 Pt 3:445–54. . 10990532 10.1111/j.1469-7793.2000.t01-1-00445.x PMC2270100

[25] Degtiar VE, Scheller RH, Tsien RW. Syntaxin modulation of slow inactivation of N-type calcium channels. J Neurosci. 2000;20(12):4355–67. . 10844004 10.1523/JNEUROSCI.20-12-04355.2000 PMC6772443

[26] Catterall WA. Structure and function of voltage-gated sodium channels at atomic resolution. Exp Physiol. 2014;99(1):35–51. Epub 2013/10/08. 10.1113/expphysiol.2013.071969 ; PubMed Central PMCID: PMCPmc3885250. 24097157 PMC3885250

[27] Silva J. Slow inactivation of Na(+) channels. Handbook of experimental pharmacology. 2014;221:33–49. Epub 2014/04/17. 10.1007/978-3-642-41588-3_3 . 24737231

[28] Kurata HT, Fedida D. A structural interpretation of voltage-gated potassium channel inactivation. Prog Biophys Mol Biol. 2006;92(2):185–208. Epub 2005/12/01. 10.1016/j.pbiomolbio.2005.10.001 . 16316679

[29] Olcese R, Latorre R, Toro L, Bezanilla F, Stefani E. Correlation between charge movement and ionic current during slow inactivation in Shaker K+ channels. J Gen Physiol. 1997;110(5):579–89. Epub 1997/11/14. ; PubMed Central PMCID: PMCPmc2229383. 9348329 10.1085/jgp.110.5.579 PMC2229383

[30] Carr DB, Day M, Cantrell AR, Held J, Scheuer T, Catterall WA, et al Transmitter modulation of slow, activity-dependent alterations in sodium channel availability endows neurons with a novel form of cellular plasticity. Neuron. 2003;39(5):793–806. Epub 2003/09/02. . 12948446 10.1016/s0896-6273(03)00531-2

[31] Chen Y, Yu FH, Surmeier DJ, Scheuer T, Catterall WA. Neuromodulation of Na+ channel slow inactivation via cAMP-dependent protein kinase and protein kinase C. Neuron. 2006;49(3):409–20. Epub 2006/02/01. 10.1016/j.neuron.2006.01.009 . 16446144

[32] Bezprozvanny I, Scheller RH, Tsien RW. Functional impact of syntaxin on gating of N-type and Q-type calcium channels. Nature. 1995;378(6557):623–6. . 8524397 10.1038/378623a0

[33] McCool BA, Pin JP, Brust PF, Harpold MM, Lovinger DM. Functional coupling of rat group II metabotropic glutamate receptors to an omega-conotoxin GVIA-sensitive calcium channel in human embryonic kidney 293 cells. Mol Pharmacol. 1996;50(4):912–22. . 8863837

[34] McCool BA, Pin JP, Harpold MM, Brust PF, Stauderman KA, Lovinger DM. Rat group I metabotropic glutamate receptors inhibit neuronal Ca2+ channels via multiple signal transduction pathways in HEK 293 cells. J Neurophysiol. 1998;79(1):379–91. . 9425207 10.1152/jn.1998.79.1.379

[35] Todd RD, McDavid SM, Brindley RL, Jewell ML, Currie KP. Gabapentin inhibits catecholamine release from adrenal chromaffin cells. Anesthesiology. 2012;116(5):1013–24. Epub 2012/03/16. 10.1097/ALN.0b013e31825153ea 22417967 PMC3341086

[36] Currie KPM, Fox AP. ATP serves as a negative feedback inhibitor of voltage-gated Ca2+ channel currents in cultured bovine adrenal chromaffin cells. Neuron. 1996;16(5):1027–36. . 8630241 10.1016/s0896-6273(00)80126-9

[37] Thaler C, Gray AC, Lipscombe D. Cumulative inactivation of N-type CaV2.2 calcium channels modified by alternative splicing. Proc Natl Acad Sci U S A. 2004;101(15):5675–9. . 15060274 10.1073/pnas.0303402101 PMC397472

[38] Swartz KJ, Merritt A, Bean BP, Lovinger DM. Protein kinase C modulates glutamate receptor inhibition of Ca2+ channels and synaptic transmission. Nature. 1993;361(6408):165–8. Epub 1993/01/14. 10.1038/361165a0 . 8380626

[39] Swartz KJ. Modulation of Ca2+ channels by protein kinase C in rat central and peripheral neurons: disruption of G protein-mediated inhibition. Neuron. 1993;11(2):305–20. Epub 1993/08/01. 0896-6273(93)90186-U [pii]. . 8102534 10.1016/0896-6273(93)90186-u

[40] Zhu Y, Ikeda SR. Modulation of Ca(2+)-channel currents by protein kinase C in adult rat sympathetic neurons. J Neurophysiol. 1994;72(4):1549–60. Epub 1994/10/01. . 7823085 10.1152/jn.1994.72.4.1549

[41] Hamid J, Nelson D, Spaetgens R, Dubel SJ, Snutch TP, Zamponi GW. Identification of an integration center for cross-talk between protein kinase C and G protein modulation of N-type calcium channels. J Biol Chem. 1999;274(10):6195–202. Epub 1999/02/26. . 10037705 10.1074/jbc.274.10.6195

[42] Barrett CF, Rittenhouse AR. Modulation of N-type calcium channel activity by G-proteins and protein kinase C. J Gen Physiol. 2000;115(3):277–86. Epub 2000/02/29. 10694257 10.1085/jgp.115.3.277 PMC2217210

[43] Diaz-Cardenas AF, Arenas I, Garcia DE. PMA counteracts G protein actions on CaV2.2 channels in rat sympathetic neurons. Arch Biochem Biophys. 2008;473(1):1–7. Epub 2008/02/27. 10.1016/j.abb.2008.01.030 . 18298939

[44] Brose N, Rosenmund C. Move over protein kinase C, you've got company: alternative cellular effectors of diacylglycerol and phorbol esters. J Cell Sci. 2002;115(Pt 23):4399–411. Epub 2002/11/05. . 12414987 10.1242/jcs.00122

[45] Yan J, Du Y, Liu J, Cao W, Sun X, Zhou W, et al Fabrication of integrated microelectrodes for electrochemical detection on electrophoresis microchip by electroless deposition and micromolding in capillary technique. Anal Chem. 2003;75(20):5406–12. Epub 2004/01/09. . 14710819 10.1021/ac034017m

[46] Daniels GM, Amara SG. Regulated trafficking of the human dopamine transporter. Clathrin-mediated internalization and lysosomal degradation in response to phorbol esters. J Biol Chem. 1999;274(50):35794–801. Epub 1999/12/10. . 10585462 10.1074/jbc.274.50.35794

[47] Ko B, Kamsteeg EJ, Cooke LL, Moddes LN, Deen PM, Hoover RS. RasGRP1 stimulation enhances ubiquitination and endocytosis of the sodium-chloride cotransporter. American journal of physiology Renal physiology. 2010;299(2):F300–9. Epub 2010/04/16. 10.1152/ajprenal.00441.2009 ; PubMed Central PMCID: PMCPmc2928521. 20392800 PMC2928521

[48] Mykoniatis A, Shen L, Fedor-Chaiken M, Tang J, Tang X, Worrell RT, et al Phorbol 12-myristate 13-acetate-induced endocytosis of the Na-K-2Cl cotransporter in MDCK cells is associated with a clathrin-dependent pathway. American journal of physiology. 2010;298(1):C85–97. Epub 2009/10/30. 10.1152/ajpcell.00118.2009 ; PubMed Central PMCID: PMCPmc2806153. 19864322 PMC2806153

[49] Kanda VA, Purtell K, Abbott GW. Protein kinase C downregulates I(Ks) by stimulating KCNQ1-KCNE1 potassium channel endocytosis. Heart rhythm: the official journal of the Heart Rhythm Society. 2011;8(10):1641–7. Epub 2011/06/28. 10.1016/j.hrthm.2011.04.034 ; PubMed Central PMCID: PMCPmc3183296. 21699843 PMC3183296

[50] Khanna R, Li Q, Bewersdorf J, Stanley EF. The presynaptic CaV2.2 channel-transmitter release site core complex. Eur J Neurosci. 2007;26(3):547–59. Epub 2007/08/10. EJN5680 [pii] 10.1111/j.1460-9568.2007.05680.x . 17686036

[51] Gandini MA, Henriquez DR, Grimaldo L, Sandoval A, Altier C, Zamponi GW, et al CaV2.2 channel cell surface expression is regulated by the light chain 1 (LC1) of the microtubule-associated protein B (MAP1B) via UBE2L3-mediated ubiquitination and degradation. Pflugers Arch. 2014;466(11):2113–26. Epub 2014/02/26. 10.1007/s00424-014-1476-4 . 24566975

[52] Simms BA, Zamponi GW. Trafficking and stability of voltage-gated calcium channels. Cell Mol Life Sci. 2012;69(6):843–56. Epub 2011/10/04. 10.1007/s00018-011-0843-y . 21964928 PMC11115007

[53] Tran-Van-Minh A, Dolphin AC. The alpha2delta ligand gabapentin inhibits the Rab11-dependent recycling of the calcium channel subunit alpha2delta-2. J Neurosci. 2010;30(38):12856–67. Epub 2010/09/24. 30/38/12856 [pii] 10.1523/JNEUROSCI.2700-10.2010 . 20861389 PMC6633565

[54] Jewell ML, Currie KPM. Control of CaV2 calcium channels and neurosecretion by heterotrimeric G protein coupled receptors In: Stephens GJ, Mochida S, editors. Modulation of presynaptic calcium channels: Springer Publishing; 2013 p. 101–30.

[55] McDavid S, Bauer MB, Brindley RL, Jewell ML, Currie KP. Butanol isomers exert distinct effects on voltage-gated calcium channel currents and thus catecholamine secretion in adrenal chromaffin cells. PLoS One. 2014;9(10):e109203 Epub 2014/10/03. 10.1371/journal.pone.0109203 ; PubMed Central PMCID: PMCPmc4183593. 25275439 PMC4183593

[56] Garcia AG, Garcia-De-Diego AM, Gandia L, Borges R, Garcia-Sancho J. Calcium signaling and exocytosis in adrenal chromaffin cells. Physiol Rev. 2006;86(4):1093–131. Epub 2006/10/04. 86/4/1093 [pii] 10.1152/physrev.00039.2005 . 17015485

[57] Wykes RC, Bauer CS, Khan SU, Weiss JL, Seward EP. Differential regulation of endogenous N- and P/Q-type Ca2+ channel inactivation by Ca2+/calmodulin impacts on their ability to support exocytosis in chromaffin cells. J Neurosci. 2007;27(19):5236–48. Epub 2007/05/15. . 17494710 10.1523/JNEUROSCI.3545-06.2007 PMC6672387

[58] Cahill AL, Hurley JH, Fox AP. Coexpression of cloned alpha(1B), beta(2a), and alpha(2)/delta subunits produces non-inactivating calcium currents similar to those found in bovine chromaffin cells. J Neurosci. 2000;20(5):1685–93. . 10684870 10.1523/JNEUROSCI.20-05-01685.2000 PMC6772916

[59] Hurley JH, Cahill AL, Currie KPM, Fox AP. The role of dynamic palmitoylation in Ca2+ channel inactivation. Proc Natl Acad Sci U S A. 2000;97(16):9293–8. . 10900273 10.1073/pnas.160589697 PMC16861

[60] Blair NT, Bean BP. Role of tetrodotoxin-resistant Na+ current slow inactivation in adaptation of action potential firing in small-diameter dorsal root ganglion neurons. J Neurosci. 2003;23(32):10338–50. Epub 2003/11/14. . 14614093 10.1523/JNEUROSCI.23-32-10338.2003 PMC6741008

[61] Choi JS, Dib-Hajj SD, Waxman SG. Differential slow inactivation and use-dependent inhibition of Nav1.8 channels contribute to distinct firing properties in IB4+ and IB4- DRG neurons. J Neurophysiol. 2007;97(2):1258–65. Epub 2006/11/17. 10.1152/jn.01033.2006 . 17108087

[62] Herlitze S, Zhong H, Scheuer T, Catterall WA. Allosteric modulation of Ca2+ channels by G proteins, voltage-dependent facilitation, protein kinase C, and Ca(v)beta subunits. Proc Natl Acad Sci U S A. 2001;98(8):4699–704. Epub 2001/04/11. doi: 10.1073/pnas.051628998 98/8/4699 [pii]. 11296298 10.1073/pnas.051628998 PMC31897

[63] Perez-Burgos A, Perez-Rosello T, Salgado H, Flores-Barrera E, Prieto GA, Figueroa A, et al Muscarinic M(1) modulation of N and L types of calcium channels is mediated by protein kinase C in neostriatal neurons. Neuroscience. 2008;155(4):1079–97. Epub 2008/07/23. 10.1016/j.neuroscience.2008.06.047 . 18644425

[64] Sculptoreanu A, de Groat WC. Protein kinase C is involved in neurokinin receptor modulation of N- and L-type Ca2+ channels in DRG neurons of the adult rat. J Neurophysiol. 2003;90(1):21–31. Epub 2003/03/28. 10.1152/jn.00108.2003 . 12660348

[65] Rajagopal S, Fang H, Oronce CI, Jhaveri S, Taneja S, Dehlin EM, et al Site-specific regulation of CA(V)2.2 channels by protein kinase C isozymes betaII and epsilon. Neuroscience. 2009;159(2):618–28. Epub 2009/01/27. S0306-4522(08)01866-6 [pii] 10.1016/j.neuroscience.2008.12.047 . 19167461

[66] Rajagopal S, Fields BL, Burton BK, On C, Reeder AA, Kamatchi GL. Inhibition of protein kinase C (PKC) response of voltage-gated calcium (Cav)2.2 channels expressed in Xenopus oocytes by Cavbeta subunits. Neuroscience. 2014;280:1–9. Epub 2014/09/15. 10.1016/j.neuroscience.2014.08.049 . 25218964

[67] Fang H, Patanavanich S, Rajagopal S, Yi X, Gill MS, Sando JJ, et al Inhibitory role of Ser-425 of the alpha1 2.2 subunit in the enhancement of Cav 2.2 currents by phorbol-12-myristate, 13-acetate. J Biol Chem. 2006;281(29):20011–7. Epub 2006/05/18. 10.1074/jbc.M601776200 . 16704976

[68] Hoshi N, Langeberg LK, Gould CM, Newton AC, Scott JD. Interaction with AKAP79 modifies the cellular pharmacology of PKC. Molecular cell. 2010;37(4):541–50. Epub 2010/03/02. 10.1016/j.molcel.2010.01.014 ; PubMed Central PMCID: PMCPmc3014287. 20188672 PMC3014287

